# Vitamin D Receptor (VDR) Polymorphisms and Cardiometabolic Profiles in Orthopedic Patients: A Cluster-Based Analysis

**DOI:** 10.3390/ijms27041958

**Published:** 2026-02-18

**Authors:** Dariusz Larysz, Remigiusz Recław, Aleksandra Suchanecka, Wojciech Dziurawiec, Rafał Tkacz, Aleksandra Strońska-Pluta, Krzysztof Chmielowiec, Anna Grzywacz, Jolanta Chmielowiec

**Affiliations:** 1 Department of Trauma and Orthopaedic Surgery, 109th Military Hospital with Polyclinic, Ministry of National Defense, ul. Ksiedza Piotra Skargi 9/11, 71-422 Szczecin, Poland; dariuszlarysz@hotmail.com (D.L.); dziurawiec.wojciech@gmail.com (W.D.); rafaltkacz@gmail.com (R.T.); 2 Independent Laboratory of Genetics and Behavioral Epigenetics, Pomeranian Medical University in Szczecin, Powstańców Wielkopolskich 72 St., 70-111 Szczecin, Poland; remigiusz.reclaw@pum.edu.pl (R.R.); aleksandra.suchanecka@pum.edu.pl (A.S.); aleksandra.stronska@pum.edu.pl (A.S.-P.); 3 Department of Medical Sciences and Public Health, Gdansk University of Physical Education and Sport, Kazimierza Górskiego 1 St., 80-336 Gdansk, Poland; 4 Department of Hygiene and Epidemiology, Collegium Medicum, University of Zielona Góra, 28 Zyty St., 65-046 Zielona Góra, Poland; chmiele@vp.pl; 5 Department of Nursing, Collegium Medicum, University of Zielona Góra, 28 Zyty St., 65-046 Zielona Góra, Poland; j.chmielowiec@inz.uz.zgora.pl

**Keywords:** vitamin D receptor (*VDR*), *COMT*, *OPRM1*, genetic polymorphisms, cardiometabolic risk, orthopedic surgery, SF-36, quality of life, cluster analysis

## Abstract

Genetic polymorphisms contribute to inter-individual variability in cardiometabolic risk and quality-of-life outcomes, yet their clinical relevance often remains unclear due to population heterogeneity and reliance on single-variant analyses. Integrative approaches combining genetic and phenotypic data may improve the characterization of complex disease profiles, particularly in orthopedic populations burdened by cardiometabolic comorbidities. This study included 289 patients scheduled for orthopedic surgery. Polymorphisms in the vitamin D receptor (*VDR*; *ApaI*, *FokI*, *BsmI*), catechol-O-methyltransferase (*COMT* rs4680), and opioid receptor mu 1 (*OPRM1* rs510769) genes were genotyped. Clinical, anthropometric, hematological, biochemical, and quality-of-life (SF-36) data were collected. Unsupervised k-means clustering was applied to standardized phenotypic variables to identify homogeneous patient subgroups. Inter-cluster differences were assessed using analysis of variance and chi-squared tests. Three distinct patient clusters were identified, characterized by specific combinations of cardiometabolic, inflammatory, and quality-of-life features. *VDR* polymorphisms were differentially distributed across clusters associated with differences in body mass index, hypertension prevalence, and inflammatory status. *COMT* and *OPRM1* variants were primarily associated with variability in physical and mental quality-of-life dimensions. The cluster-based approach revealed multidimensional clinical heterogeneity not captured by conventional univariate analyses. Integrating genetic polymorphisms with clinical and quality-of-life data may support the identification and interpretation of distinct cardiometabolic profiles among orthopedic patients. Cluster-based stratification represents a valuable framework for capturing complex patient heterogeneity and supports future precision-oriented research in orthopedic and cardiometabolic populations.

## 1. Introduction

Inter-individual variability in disease susceptibility, clinical presentation, and treatment response represents a major challenge in contemporary medicine [1]. Increasing evidence indicates that genetic polymorphisms contribute substantially to phenotypic heterogeneity observed across a wide range of chronic and multifactorial diseases [2,3]. Single-nucleotide polymorphisms may influence inflammatory pathways, metabolic regulation, and cardiometabolic risk, thereby shaping complex clinical profiles rather than isolated disease traits [4]. Consequently, integrative research approaches that combine genetic variation with clinical and biological characteristics are increasingly required to better capture the multidimensional nature of disease-related heterogeneity [5].

The vitamin D receptor (*VDR*) is a key mediator of vitamin D-dependent signaling and plays an essential role in regulating gene expression across multiple tissues [6]. Beyond its established involvement in bone metabolism, *VDR* activity has been linked to immune modulation, inflammatory processes, glucose homeostasis, and blood pressure regulation [7]. Common polymorphisms within the *VDR* gene, including *ApaI*, *FokI*, and *BsmI*, have therefore attracted considerable attention in studies exploring metabolic and cardiovascular conditions [8]. However, findings across these studies have often been inconsistent, suggesting that the clinical relevance of *VDR* polymorphisms may depend on broader cardiometabolic and biological contexts rather than on single phenotypic outcomes [9,10].

In addition to *VDR*, polymorphisms in genes involved in neurotransmission and stress regulation may further contribute to inter-individual differences in clinical and quality-of-life outcomes [11]. The catechol-O-methyltransferase (*COMT*) gene plays a key role in catecholamine metabolism and has been associated with pain perception, stress responsivity, and cardiometabolic regulation [12]. Similarly, variation within the opioid receptor mu 1 (*OPRM1*) gene has been linked to pain processing, inflammatory responses, and behavioral factors relevant to clinical outcomes [13]. Including these genes allows for a broader exploration of molecular factors potentially underlying heterogeneous cardiometabolic and quality-of-life profiles [14]. Together, these genes were examined to capture complementary cardiometabolic and psychosocial mechanisms potentially shaping complex clinical profiles in orthopedic patients.

Most existing studies examining genetic polymorphisms have focused on associations between individual variants and single clinical endpoints, often yielding heterogeneous or contradictory results [15,16]. Such reductionist approaches may be insufficient to capture the complex interplay between genetic variation and multidimensional clinical phenotypes [17]. Moreover, orthopedic patient populations–typically characterized by advanced age and a high burden of cardiometabolic comorbidities–remain relatively underexplored in integrative genetic research [18].

Nevertheless, there is limited understanding of how genetic polymorphisms contribute to multidimensional clinical heterogeneity in orthopedic patients [19]. Given the cardiometabolic burden typical of orthopedic populations, we focused on routinely available hematological and biochemical parameters reflecting key perioperative health domains, including inflammatory status (CRP and leukocyte subpopulations), metabolic regulation (HbA1c), renal function (creatinine), and vitamin D status (25-hydroxyvitamin D_3_). Together with anthropometric measures and SF-36 quality-of-life scores, these variables were selected to capture clinically meaningful multidimensional heterogeneity in this patient cohort. In this context, previous studies in orthopedic patients have demonstrated that individual *VDR* polymorphisms, such as rs2228570 (*FokI*) and rs7975232 (*ApaI*), may be associated with inflammatory markers and patient-reported quality-of-life outcomes [20,21]. However, these hypothesis-driven, single-variant analyses do not capture the multidimensional cardiometabolic and psychosocial complexity observed in this clinical population, underscoring the need for integrative, data-driven approaches.

Unsupervised clustering methods provide a data-driven framework for identifying homogeneous subgroups within clinically heterogeneous populations [22,23]. By simultaneously incorporating clinical, biochemical, and quality-of-life variables, cluster-based approaches enable the detection of latent patient profiles that may not be apparent using conventional univariate analyses [24]. Such multidimensional stratification may offer valuable insights into how genetic polymorphisms interact with cardiometabolic and clinical characteristics, thereby facilitating a more nuanced interpretation of complex disease phenotypes [25]. In this context, clustering techniques represent a promising tool for integrative molecular and clinical investigations [26].

The aim of the present study was to analyze vitamin D receptor (*VDR*) polymorphisms in relation to cardiometabolic and quality-of-life profiles in patients undergoing orthopedic surgery. We therefore applied an unsupervised k-means clustering framework to identify naturally emerging patient subgroups based on multidimensional phenotypic variables (clinical, laboratory, and quality-of-life characteristics) [27]. This exploratory, hypothesis-generating strategy was used to characterize multivariate heterogeneity without imposing a priori assumptions about linearity or predefined outcome–predictor structures. *VDR* polymorphisms were subsequently examined in relation to the identified phenotype-based clusters.

## 2. Results

This section presents the results of clinical, laboratory, and genetic analyses performed in the orthopedic patient cohort. First, baseline clinical characteristics of the study population are described to provide context for molecular and statistical analyses. This is followed by the characterization of biochemical and quality-of-life parameters, the distribution of selected genetic polymorphisms, and the identification of distinct patient subgroups using cluster analysis, highlighting differences in demographic, clinical, laboratory, and quality-of-life profiles.

The study population consisted of 289 participants, with women constituting the majority of the cohort. Most participants were non-smokers, while hypertension was highly prevalent, affecting approximately two thirds of the cohort. Diabetes mellitus was less common, being reported by fewer than one fifth of participants, indicating a cardiometabolic risk profile dominated primarily by hypertension rather than overt diabetes (Table 1).

Table 2 summarizes the descriptive statistics of demographic, anthropometric, biochemical, and quality-of-life variables in the orthopedic patient cohort (N = 289). The study population consisted predominantly of older adults, with a generally elevated body mass index indicative of overweight status. Mean BMI indicated obesity at the cohort level, reflecting a high cardiometabolic burden in this orthopedic population. Hematological parameters, including leukocyte subpopulations, erythrocyte indices, and platelet-related measures, were largely within clinically expected ranges, although substantial inter-individual variability was observed for several variables.

Inflammatory and metabolic markers demonstrated pronounced dispersion, particularly C-reactive protein, vitamin D_3_ concentration, HbA1c, and creatinine levels, reflecting heterogeneity in inflammatory burden, metabolic regulation, and renal function. Vitamin D_3_ concentrations showed a wide range, including extreme values, reflecting real-world variability in deficiency and supplementation patterns. Assessment of health-related quality of life using the SF-36 questionnaire revealed moderate physical and mental health scores, with wide ranges indicating considerable variability in patient-reported outcomes.

Sex-related differences in phenotypic variables are presented in Table 3. Compared with women, men showed significantly higher hemoglobin, hematocrit, and erythrocyte-related indices (MCV, MCH, and MCHC), as well as higher creatinine levels, reflecting expected sex-related physiological differences. Platelet count and plateletcrit were significantly lower in men. In contrast, women presented significantly higher serum 25-hydroxyvitamin D concentrations. No significant differences were observed between sexes in SF-36 physical and mental component summary scores. These sex-related differences were taken into account when interpreting cluster profiles.

Given the observed clinical and biochemical heterogeneity within the cohort, the distribution of selected genetic polymorphisms was subsequently examined to provide a genetic context for the identified variability. Genotype distributions for the rs7975232 (*ApaI*), rs2228570 (*FokI)*, rs1544410 (*BsmI*), rs4680 (*COMT*) and rs510769 (*OPRM1)* polymorphisms conformed to Hardy–Weinberg equilibrium (Table 4).

The distribution of genotypes for the analyzed single-nucleotide polymorphisms is presented in Table 4. For rs7975232 (*ApaI*), rs2228570 (*FokI*), rs1544410 (*BsmI*), and rs4680 (*COMT*), the heterozygous genotype was the most frequently observed across the study population. In contrast, the rs510769 (*OPRM1*) polymorphism was characterized by a predominance of the homozygous wild-type genotype. These distributions provided an appropriate basis for subsequent post hoc comparisons of genotype frequencies across the phenotype-based clusters.

Table 5 presents the distribution of observations across three clusters together with the standardized distances between cluster centroids. Cluster 1 included 95 observations (32.87%), Cluster 2 comprised 119 observations (41.18%), and Cluster 3 contained 75 observations (25.95%). Pairwise distances between cluster centroids are shown, with diagonal values equal to zero. Identical distances observed between all cluster pairs (2.01) indicate a comparable degree of separation among the identified clusters.

**Figure 1:**
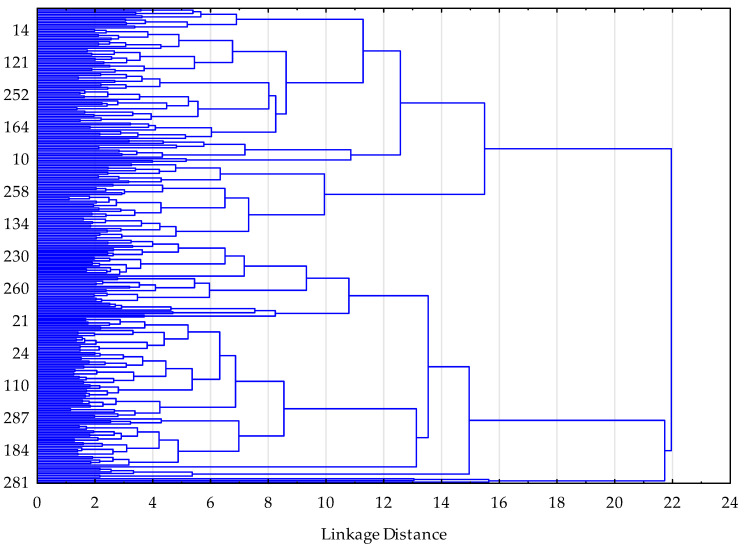
Dendrogram from hierarchical cluster analysis illustrating linkage distances between observations based on standardized phenotypic variables (clinical, laboratory, and SF-36 quality-of-life measures). A pronounced increase in linkage distance when moving from three to two clusters supports the selection of a three-cluster solution (k = 3) according to the elbow criterion.

Following the identification of three distinct clusters based on hierarchical clustering and the elbow criterion (Table 5, Figure 1), between-cluster comparisons were performed to examine differences in clinical, laboratory, and quality-of-life characteristics.

Table 6 summarizes the comparison of demographic and clinical characteristics across the three identified clusters. Sex distribution differed significantly between clusters (χ^2^ = 87.92, *p* < 0.001), with Cluster 2 being predominantly female, whereas Clusters 1 and 3 showed a more balanced or male-dominated distribution. Smoking status also varied significantly across clusters (χ^2^ = 12.37, *p* = 0.002), with the highest proportion of smokers observed in Cluster 1 and the lowest in Cluster 2. Hypertension demonstrated a strong association with cluster membership (χ^2^ = 70.31, *p* < 0.001), being most prevalent in Clusters 1 and 2 and markedly less frequent in Cluster 3. In contrast, no statistically significant differences were observed between clusters with respect to diabetes mellitus (χ^2^ = 5.43, *p* = 0.066), although a trend toward variation was noted.

Following the observed differences in demographic and clinical characteristics across clusters, continuous anthropometric, hematological, biochemical, and quality-of-life parameters were subsequently compared between the identified clusters.

Table 7 presents the comparison of continuous anthropometric, hematological, biochemical, and quality-of-life parameters across the three identified clusters. Significant inter-cluster differences were observed for age and body mass index, with Cluster 3 comprising younger participants and exhibiting the lowest BMI, while Cluster 1 showed the highest BMI values.

Several hematological parameters differed significantly between clusters, including white blood cell, neutrophil, monocyte, and basophil counts. Differences were also observed for red blood cell indices, such as hemoglobin, hematocrit, mean corpuscular volume, mean corpuscular hemoglobin, and mean corpuscular hemoglobin concentration, with generally higher values in Cluster 3. Platelet count and plateletcrit varied significantly across clusters, whereas platelet distribution width and mean platelet volume did not.

No significant inter-cluster differences were observed in inflammatory markers, vitamin D status, glycated hemoglobin, or creatinine levels. In contrast, quality-of-life assessment revealed significant inter-cluster variation, with the highest physical component summary (PCS) scores observed in Cluster 2 and the highest mental component summary (MCS) scores in Cluster 1.

Given the significant association between sex and cluster membership observed in Table 6, a sex-stratified analysis was subsequently performed to evaluate whether inter-cluster differences remained consistent within women and men separately (Table 8).

In women (see Table 8), significant inter-cluster differences were observed for age (F_2,176_ = 4.00; *p* = 0.020) and body mass index (BMI) (F_2,176_ = 4.08; *p* = 0.018). A significant difference was also found for lymphocyte count (LYM) (F_2,176_ = 7.80; *p* < 0.001), which remained significant after Bonferroni correction.

Regarding biochemical and quality-of-life parameters, clusters differed significantly in creatinine concentration (F_2,176_ = 3.73; *p* = 0.026) and in both components of the SF-36 questionnaire: the physical component summary (PCS) (F_2,176_ = 5.22; *p* = 0.006) and the mental component summary (MCS) (F_2,176_ = 5.15; *p* = 0.007). No significant inter-cluster differences were observed for the remaining hematological, biochemical, or platelet-related parameters (*p* > 0.05).

In men, significant inter-cluster differences were detected for age (F_2,107_ = 4.20; *p* = 0.018) and BMI (F_2,107_ = 3.75; *p* = 0.027). Among hematological parameters, significant differences were observed for monocyte count (MONO) (F_2,107_ = 6.24; *p* = 0.003) and platelet distribution width (PDW) (F_2,107_ = 3.61; *p* = 0.030). Clusters also differed significantly in creatinine concentration (F_2,107_ = 3.85; *p* = 0.025). In the quality-of-life analysis, a significant difference was identified for the mental component summary (MCS) (F_2,107_ = 3.19; *p* = 0.045), whereas the physical component summary (PCS) did not reach statistical significance (*p* = 0.067). No other parameters differed significantly between clusters (*p* > 0.05).

Overall, sex-stratified analysis confirmed the stability of the previously identified clusters and did not undermine the validity of the original cluster solution. Key inter-cluster differences observed in the full-sample analysis–particularly for age, BMI, and selected SF-36 dimensions–remained partially evident after stratification. However, the magnitude and pattern of differences varied between women and men, indicating a modulatory rather than dominant role of biological sex. Specifically, in women, clusters were primarily differentiated by quality-of-life parameters and lymphocyte count, whereas in men, differences were more pronounced for monocytes and platelet-related indices. These findings suggest that sex does not constitute the principal determinant of cluster membership but influences the phenotypic expression of cluster-specific profiles.

Given the distinct multidimensional profiles observed across clusters, genotype distributions of selected single-nucleotide polymorphisms were subsequently compared post hoc to examine their associations with phenotype-based cluster membership (Table 9).

As shown in Table 9, genotype distributions differed across the three identified clusters. Statistically significant differences were observed for rs7975232 (*ApaI*), rs2228570 (*FokI*), rs1544410 (*BsmI*), and rs510769 (*OPRM1*), indicating distinct genotype frequencies across the phenotype-based clusters. In contrast, no significant inter-cluster differences were detected for the rs4680 polymorphism in the *COMT* gene, suggesting a comparable genotype distribution across clusters for this variant.

**Figure 2:**
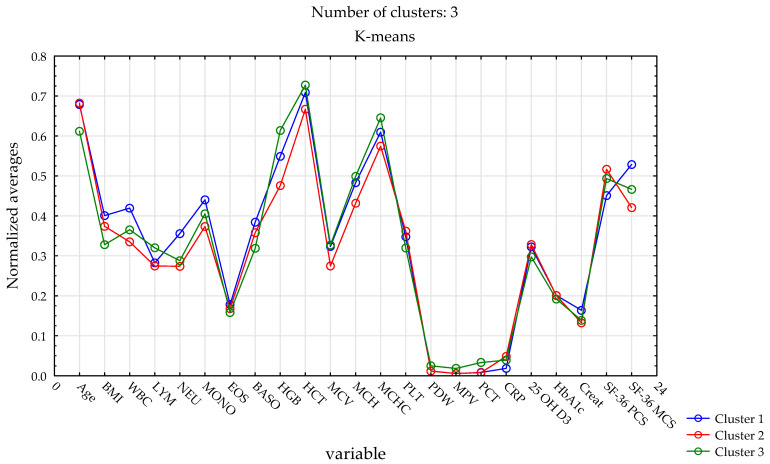
Comparison of the three identified clusters in terms of selected clinical, hematological, biochemical parameters and SF-36 quality-of-life scores.

K-means clustering (k = 3; total n = 289) identified three distinct phenotype-based patient clusters differing in demographic, clinical, laboratory, and quality-of-life characteristics (Figure 2). Cluster 1 included a higher proportion of men and was characterized by higher white blood cell and neutrophil counts, higher body mass index, and relatively higher mental health–related quality-of-life scores. Cluster 2, composed mainly of women, exhibited intermediate clinical and laboratory profiles, with values generally falling between those observed in Clusters 1 and 3, and the highest physical quality-of-life scores. Cluster 3 comprised younger participants with lower body mass index, distinct genotype distributions, higher hemoglobin and hematocrit values, and a lower prevalence of hypertension. Genotype distributions were subsequently compared post hoc across the identified phenotype-based clusters (Table 9), providing additional genetic context for the observed phenotypic profiles.

## 3. Discussion

The present study demonstrated that integrating genetic polymorphisms with clinical, laboratory, and quality-of-life data may support the identification and interpretation of distinct cardiometabolic profiles among orthopedic patients. Using an unsupervised cluster-based approach, we identified heterogeneous phenotype-based patient subgroups characterized by distinct combinations of anthropometric, clinical, laboratory, and quality-of-life features. Genotype distributions for *VDR*, *COMT*, and *OPRM1* polymorphisms were subsequently examined post hoc across the identified clusters, providing additional genetic context for the observed phenotypic variability. Importantly, this integrative framework extends beyond traditional single-variant analyses and highlights the value of data-driven stratification in capturing potentially biologically and clinically meaningful differences within a seemingly homogeneous orthopedic population.

In the present study, polymorphisms within the vitamin D receptor (*VDR*) gene emerged as important components of the multidimensional cardiometabolic profiles identified by cluster analysis. Variants of *ApaI*, *FokI*, and *BsmI* were differentially distributed across clusters characterized by distinct patterns of body mass index, hypertension prevalence, and inflammatory markers, supporting the notion that *VDR*-related effects are context dependent rather than uniform across individuals [6,7,8,9,10,22]. These observations are consistent with the view that *VDR* polymorphisms do not exert isolated effects on single cardiometabolic traits, but instead contribute to broader phenotypic configurations shaped by interactions between metabolic, inflammatory, and clinical factors. Importantly, the cluster-based approach applied in this study offers a potential framework for reconciling previously inconsistent findings regarding VDR polymorphisms by demonstrating that their clinical relevance may become apparent only within specific patient subgroups defined by integrated cardiometabolic characteristics [28,29,30,31].

Beyond cardiometabolic characteristics, polymorphisms in genes related to neurotransmission and stress regulation were examined in relation to cluster-specific quality-of-life profiles [32,33,34,35]. Although *COMT* rs4680 genotype frequencies did not differ significantly across clusters, this variant remains biologically relevant due to its role in catecholamine metabolism and has been linked to stress responsivity and psychological functioning in prior studies. In contrast, variation in the opioid receptor mu 1 (*OPRM1*) gene showed significant differences across the identified phenotype-based clusters, and may relate to inter-individual variability in pain perception, stress processing, and behavioral responses relevant to orthopedic patients. Together, these findings indicate that *COMT* and *OPRM1* polymorphisms contribute primarily to neurobiological and psychosocial dimensions of patient heterogeneity, complementing the cardiometabolic patterns associated with *VDR* polymorphisms and underscoring the value of integrating genetic data with quality-of-life measures [32,33,34,35].

The use of unsupervised cluster analysis represents a key methodological strength of the present study, as it enables the identification of potentially clinically meaningful patient subgroups without imposing predefined assumptions regarding outcome variables [36,37,38,39,40,41]. Although not intended for direct clinical decision-making, the identified clusters highlight patterns that may support clinicians and researchers in recognizing distinct patient profiles. By integrating multidimensional phenotypic variables in the clustering stage and subsequently examining genetic polymorphisms post hoc, this approach captures the multidimensional nature of patient heterogeneity more effectively than traditional univariate or single-variant analyses. This data-driven stratification shifts the analytical focus from isolated associations toward patient-level profiles, thereby reflecting the complex interplay between molecular and phenotypic factors observed in real-world clinical populations [36,37,38,39,40,41]. In the context of orthopedic patients, who often present with overlapping cardiometabolic, inflammatory, and psychosocial characteristics, such an approach provides a robust framework for uncovering latent structures that may otherwise remain obscured [37,40,41].

From a clinical perspective, the identification of distinct cardiometabolic and quality-of-life profiles among orthopedic patients highlights the heterogeneity that exists within populations often considered relatively uniform in routine practice. The observed cluster-specific patterns suggest that genetic variation, when considered alongside clinical and laboratory parameters, may help to contextualize differences in cardiometabolic burden, inflammatory status, and patient-reported outcomes [42,43,44]. Although the present findings do not support the use of individual genetic variants as standalone clinical markers, they underscore the potential value of integrative stratification approaches for improving the characterization of patient subgroups with differing clinical needs [45]. Such multidimensional profiling may inform future research aimed at refining risk assessment, optimizing perioperative management, and tailoring supportive interventions in orthopedic populations while acknowledging that further validation in independent cohorts is required [42].

Several limitations of the present study should be acknowledged. First, the absence of a non-orthopedic control group limits the ability to directly compare genotype distributions and clinical profiles with those of the general population. Genotype distributions were consistent with the Hardy–Weinberg equilibrium within the present cohort; however, replication in independent populations is required. Second, the observational and cross-sectional design restricts causal inference regarding the relationships between genetic polymorphisms and cardiometabolic or quality-of-life characteristics. Third, although the sample size was sufficient for cluster analysis, this study was conducted in a single clinical center, which may limit the generalizability of the findings. The predominance of women may limit the generalizability to male orthopedic patients and should be considered when interpreting subgroup distributions. Additionally, the analyzed polymorphisms were not complemented by functional assays, preventing direct conclusions regarding their biological effects. Finally, residual confounding by unmeasured lifestyle or environmental factors cannot be excluded. These limitations should be considered when interpreting the results and highlight the need for replication in larger, multicenter cohorts with longitudinal designs.

## 4. Materials and Methods

### 4.1. Participants

This study included 289 patients scheduled for elective orthopedic surgery. The cohort comprised 110 men (38.06%) and 179 women (61.94%), with a mean age of 66.7 years. Participants were recruited from the Department of Orthopaedics of the 109th Military Hospital with Polyclinic in Szczecin, Poland. Inclusion criteria were: age ≥ 18 years; eligibility for knee or hip replacement surgery or spinal surgery for degenerative disease; and provision of written informed consent prior to participation. Exclusion criteria included active malignancy; infectious or autoimmune disease; pregnancy or breastfeeding; a diagnosis of intellectual disability or dementia; and clinically significant general health conditions (e.g., cardiovascular, liver, kidney, respiratory, hematological, endocrinological, or neurological disease) or any clinically significant laboratory abnormality. This was a single-center cross-sectional observational study.

### 4.2. Measures

Blood samples were collected between May 2023 and May 2025 from all participants for laboratory assessment of hematological, inflammatory, and metabolic markers. The analyzed parameters included leukocyte count and differential (white blood cells, lymphocytes, neutrophils, monocytes, eosinophils, and basophils), red blood cell indices (hemoglobin concentration, hematocrit, mean corpuscular volume, mean corpuscular hemoglobin, and mean corpuscular hemoglobin concentration), and platelet-related measures (platelet count, platelet distribution width, mean platelet volume, and plateletcrit). In addition, concentrations of C-reactive protein, serum 25-hydroxyvitamin D_3_, glycated hemoglobin, and creatinine were determined.

Hematological parameters were measured using a Sysmex XN-550 hematology analyzer (Sysmex Corporation, Kobe, Japan). Biochemical parameters (CRP, creatinine, and HbA1c) were measured on the cobas pure platform using the biochemical module cobas c 303 (Roche Diagnostics GmbH, Mannheim, Germany). Serum total 25-hydroxyvitamin D (25-OH-D) was measured using the immunochemistry module cobas e 402 on the cobas pure platform (Roche Diagnostics). According to internal laboratory quality control, the inter-assay coefficient of variation (CV) was 8.64% at the low control level and 7.35% at the high control level.

Body mass index (BMI) was calculated for each participant as body weight expressed in kilograms divided by height in meters squared.

Health-related quality of life was assessed using the Short Form Health Survey (SF-36), a standardized 36-item questionnaire covering eight domains, including physical functioning, role limitations related to physical and emotional health, bodily pain, general health perception, vitality, social functioning, and mental health.

### 4.3. Genotyping

Genomic DNA was isolated from peripheral venous blood using standard procedures in accordance with the manufacturer’s instructions (Roche, Basel, Switzerland). Genotyping of all analyzed single-nucleotide polymorphisms was performed using real-time polymerase chain reaction with allele-specific fluorescent probes and melting curve analysis, employing TIB MOLBIOL LightSNiP assays on a Roche LightCycler 480 system (Roche Diagnostics GmbH, Mannheim, Germany). Negative (no-template) controls were included in each analytical run, and a subset of samples was re-genotyped to verify analytical reliability.

Allelic discrimination was based on characteristic melting temperatures. For vitamin D receptor (*VDR*) polymorphisms, melting peaks were observed at approximately 62.8 °C (C allele) and 69.9 °C (A allele) for rs7975232 (*ApaI*), at 57.5 °C (C allele) and 63.8 °C (T allele) for rs2228570 (*FokI*), and at 56.5 °C (A allele) and 66.3 °C (G allele) for rs1544410 (*BsmI*). For the catechol-O-methyltransferase (*COMT*) rs4680 (Val158Met) polymorphism, peaks occurred at approximately 59.9 °C for the G (Val) allele and 53.3 °C for the A (Met) allele. The opioid receptor mu 1 (*OPRM1*) rs510769 polymorphism was distinguished by melting peaks at approximately 53.2 °C for the C allele and 57.8 °C for the T allele.

### 4.4. Statistics

Hardy–Weinberg equilibrium (HWE) was assessed for genotype distributions using an online calculator (https://wpcalc.com/en/equilibrium-hardy-weinberg/ (accessed on 1 January 2026)). The distribution of continuous variables was examined using quantile–quantile (Q–Q) plots and the Kolmogorov–Smirnov test. When deviations from normality were observed, logarithmic transformation was applied prior to further analyses. Variables meeting assumptions of normality are presented as mean ± standard deviation (SD), whereas variables with non-normal distributions are reported as median and interquartile range (Q1–Q3). Differences in blood parameters between genders were assessed using Student’s *t*-test for normally distributed variables and the Mann–Whitney U test for non-normally distributed variables. For categorical variables, differences were evaluated using the chi-squared test.

Descriptive statistics for biochemical parameters and SF-36 quality-of-life scores included measures of central tendency and dispersion, as well as 95% confidence intervals. Genotype data are presented as absolute frequencies and percentages.

Formal a priori power calculation was not performed because the clustering stage was exploratory; instead, statistical sensitivity was evaluated using observed effect sizes from subsequent between-cluster comparisons.

The clustering analysis was performed to capture multidimensional phenotypic heterogeneity and to derive internally coherent patient profiles without assuming linearity or specifying predefined outcome–predictor relationships. Thus, unsupervised clustering was selected to address a different aim than classical multivariable regression approaches.

While classical multivariate models aim to estimate independent effects of individual variables, the objective of the present study was to identify multivariate patient profiles, which justified the use of a clustering approach. A two-stage clustering procedure was applied. In the first stage, hierarchical clustering was conducted as an exploratory step to determine the optimal number of clusters. Visual inspection of the dendrogram and the agglomeration schedule revealed a marked increase in linkage distance when moving from three to two clusters, supporting a three-cluster solution (k = 3) based on the elbow criterion. In the second stage, k-means clustering using Euclidean distance was performed in STATISTICA, with a maximum of 50 iterations and an initialization procedure designed to maximize the distance between cluster centroids. Cluster stability was evaluated indirectly by examining the consistency of cluster solutions across repeated algorithm iterations. First, clustering was conducted in the entire cohort; subsequently, separate clustering analyses were performed within women and men to assess the stability of the identified structure.

Clustering was performed exclusively using quantitative clinical and laboratory variables. Genetic polymorphisms were not included in the clustering input and were examined only post hoc across the derived phenotype-based clusters. Post hoc differences in continuous variables between clusters were evaluated using one-way analysis of variance (ANOVA) with Welch’s correction for unequal variances, whereas categorical variables, including genotype frequencies, were compared using the chi-squared test. Post hoc pairwise comparisons were adjusted using the Bonferroni correction. Statistical significance was defined as a two-sided *p*-value < 0.05. All analyses were conducted using STATISTICA version 13 (Tibco Software Inc., Palo Alto, CA, USA) and PQStat version 1.8.6 (PQStat Software, Poznań, Poland) for Windows 11 Pro.

## 5. Conclusions

In conclusion, this study demonstrates that integrating genetic polymorphisms with clinical, biochemical, and quality-of-life data may support the identification and interpretation of distinct cardiometabolic profiles among orthopedic patients. The identified phenotype-based subgroups were characterized by unique combinations of cardiometabolic burden, inflammatory status, and patient-reported outcomes. *VDR* and OPRM1 polymorphisms were subsequently compared post hoc across the identified clusters, providing additional genetic context for the observed phenotypic heterogeneity. The application of unsupervised cluster analysis highlights the value of data-driven stratification for capturing multidimensional clinical heterogeneity beyond traditional single-variant approaches. Although further validation is required, this integrative framework provides a foundation for future studies aimed at improving phenotypic stratification and advancing precision-oriented research in orthopedic and cardiometabolic populations. Together with our previous hypothesis-driven analyses of individual *VDR* polymorphisms in orthopedic patients [20,21], the present cluster-based study contributes to a broader, integrative framework for understanding genetic and cardiometabolic heterogeneity in this clinical population.

## Figures and Tables

**Table 1:** Clinical characteristics of the orthopedic patient cohort (N = 289).

Variable	Yes n (%)	No n (%)
Smoking status	31 (10.73%)	258 (89.27%)
Hypertension	191 (66.09%)	98 (33.91%)
Diabetes mellitus	57 (19.72%)	232 (80.28%)

n—number of subjects, data are presented as n (% of the total sample).

**Table 2:** Descriptive statistics of biochemical parameters and SF-36 quality of life scores in a cohort of 289 orthopedic patients.

Variable	M	95% CI (Lower)	95% CI (Upper)	Median	Q1	Q3	Minimum	Maximum	SD	Variation Factor	Standard Error
Age	66.75	65.58	67.92	68.00	62.00	73.00	27.00	87.00	10.08	15.15	0.59
Body mass index (BMI)	29.22	28.64	29.83	28.72	25.74	31.79	17.93	48.42	5.05	17.33	0.30
White blood cells (WBC, ×10^9^/L)	6.98	6.75	7.21	6.74	5.58	8.25	2.40	14.7	1.97	28.28	0.12
Lymphocytes (LYM, ×10^9^/L)	1.70	1.64	1.77	1.65	1.32	2.00	0.60	4.42	0.57	33.50	0.03
Neutrophils (NEU, ×10^9^/L)	4.57	4.38	4.77	4.38	3.36	5.39	1.13	12.44	1.69	37.15	0.10
Monocytes (MONO, ×10^9^/L)	0.53	0.51	0.55	0.51	0.39	0.63	0.05	1.23	0.17	33.03	0.01
Eosinophils (EOS, ×10^9^/L) ǂ*	0.12	0.11	0.13	0.09	0.05	0.17	0.00	0.73	0.10		0.01
Basophils (BASO, ×10^9^/L) ǂ	0.04	0.03	0.04	0.03	0.02	0.05	0.00	0.10	0.02		0.001
Hemoglobin (HGB, g/dL)	13.80	13.65	13.98	13.80	13.10	14.80	9.10	17.90	1.44	10.40	0.08
Hematocrit (HCT, %)	41.20	40.71	41.73	41.20	38.90	44.10	14.70	52.80	4.40	10.69	0.26
Mean corpuscular volume (MCV, fL)	90.58	90.08	91.24	90.90	87.40	93.30	76.500	120.30	5.08	5.54	0.30
Mean corpuscular hemoglobin (MCH, pg)	30.18	29.97	30.45	30.30	29.10	31.40	20.80	41.00	2.13	6.95	0.12
Mean corpuscular hemoglobin concentration (MCHC, g/dL)	33.35	33.24	33.47	33.30	32.80	33.90	28.40	36.60	0.97	2.88	0.06
Platelet count (PLT, ×10^9^/L)	256.89	247.87	264.50	252.00	210.00	294.00	12.00	717.00	71.87	28.03	4.22
Platelet distribution width (PDW, %) ǂ	12.27	12.03	12.52	11.90	10.90	13.50	8.70	24.90	2.10		0.12
Mean platelet volume (MPV, fL) ǂ	10.46	10.35	10.57	10.40	9.80	11.10	8.60	14.70	0.95		0.06
Plateletcrit (PCT, %) ǂ	0.43	0.24	0.62	0.26	0.22	0.31	0.09	0.68	0.63		0.09
C-reactive protein (CRP, mg/L) ǂ	7.77	5.04	10.49	2.01	0.99	4.49	0.05	211.55	23.54		1.38
25-hydroxyvitamin D3 (ng/mL) ǂ	32.39	30.52	34.26	28.85	20.40	40.70	0.35	101.00	16.16		0.95
Glycated hemoglobin (HbA1c, %) ǂ	5.76	5.55	5.96	5.63	5.38	6.00	4.72	9.85	1.78		0.10
Creatinine (µmol/L) ǂ	80.041	76.9911	83.0791	73.50	65.00	86.50	46.00	282.00	26.11		1.55
SF-36 PCS	45.67	44.42	46.69	45.67	39.76	51.47	16.37	76.08	10.67	21.48	0.58
SF-36 MCS	47.83	46.21	49.44	47.83	37.98	57.07	6.69	94.60	14.89	29.16	0.82

ǂ—variables do not follow a normal distribution according to the Kolmogorov–Smirnov test; n—number of subjects; *—statistically significant difference, *p* < 0.05; M—mean; CI—confidence interval, −95% CI; +95% CI; Q1—lower quartile; Q3—upper quartile; SD—standard deviation.

**Table 3:** Descriptive statistics of biochemical parameters and SF-36 quality of life scores in a cohort of women and men orthopedic patients.

	Women n = 179	Men n = 110		Student’s *t*-Test/ Mann–Whitney		
Variable	M	SD	M	SD		*p*
Age	67.02	10.38	66.31	9.69	−0.57	0.566
Body mass index (BMI)	28.78	5.00	29.97	5.07	1.94	0.053
White blood cells (WBC, ×10^9^/L)	6.82	2.01	7.25	1.90	1.81	0.072
Lymphocytes (LYM, ×10^9^/L)	1.69	0.58	1.73	0.55	0.59	0.553
Neutrophils (NEU, ×10^9^/L)	4.46	1.73	4.75	1.63	1.39	0.166
Monocytes (MONO, ×10^9^/L)	0.50	0.17	0.57	0.18	3.87	<0.001 *#
Eosinophils (EOS, ×10^9^/L) ǂ	0.12	0.11	0.13	0.10	1.27	0.202
Basophils (BASO, ×10^9^/L) ǂ	0.04	0.02	0.04	0.02	−0.77	0.443
Hemoglobin (HGB, g/dL)	13.37	1.20	14.53	1.50	7.24	<0.001 *#
Hematocrit (HCT, %)	40.07	4.15	43.11	4.17	6.03	<0.001 *#
Mean corpuscular volume (MCV, fL)	90.02	4.93	91.69	5.03	2.77	0.006 *
Mean corpuscular hemoglobin (MCH, pg)	29.79	2.12	30.90	1.88	4.52	<0.001 *#
Mean corpuscular hemoglobin concentration (MCHC, g/dL)	33.14	0.95	33.70	0.88	4.99	<0.001 *#
Platelet count (PLT, ×10^9^/L)	266.84	76.46	238.84	59.92	−3.27	0.001 *#
Platelet distribution width (PDW, %) ǂ	12.39	2.19	12.11	1.97	−1.10	0.271
Mean platelet volume (MPV, fL) ǂ	10.50	1.00	10.39	0.89	−0.79	0.428
Plateletcrit (PCT, %) ǂ	0.36	0.95	0.54	2.35	−3.87	<0.001 *#
C-reactive protein (CRP, mg/L) ǂ	7.85	22.47	7.64	25.29	−0.97	0.331
25-hydroxyvitamin D3 (ng/mL) ǂ	34.45	16.41	29.02	15.21	−3.21	0.001 *#
Glycated hemoglobin (HbA1c, %) ǂ	5.80	2.06	5.68	1.18	0.53	0.595
Creatinine (µmol/L) ǂ	74.06	23.50	89.68	27.30	6.29	<0.001 *#
SF-36 PCS	45.61	9.97	45.46	9.52	−0.13	0.899
SF-36 MCS	47.11	13.85	48.99	14.10	1.11	0.266

ǂ—variables do not follow a normal distribution according to the Kolmogorov–Smirnov test. Student’s *t*-test—applied for variables with a normal distribution; Mann–Whitney U test—applied for variables without a normal distribution; *—statistically significant difference, *p* < 0.05; #—Bonferroni correction: the *p*-value threshold was lowered to 0.002 (0.05/23 = 0.0022).

**Table 4:** Distribution of genotypes for selected single-nucleotide polymorphisms in the study population and Hardy–Weinberg equilibrium (HWE) analysis for rs7975232 (*ApaI*), rs2228570 (*FokI*), rs1544410 (*BsmI*), rs4680 (*COMT*), and rs510769 (*OPRM1*).

**Variable**	**Homozygous Wild n (%)**		**Heterozygous** **N (%)**		**Homozygous** **Recessive n (%)**		**Major** **Allele**	**Minor Allele**	**Hardy–Weinberg Equilibrium**	
	observed	expected	observed	expected	observed	expected			Χ^2^	*p*
rs7975232 *ApaI*	C/C		C/A		A/A		C	A		
	73 (25.26%)	74.8	148 (51.21%)	144.5	68 (23.53%)	69.8	294 (51.86%)	284 (49.14%)	0.17	0.677
rs2228570 *FokI*	C/C		C/T		T/T		C	T		
	94 (32.53%)	95.9	145 (50.17%)	141.2	50 (17.30%)	51.9	333 (57.61%)	245 (42.39%)	0.22	0.643
rs1544410 *BsmI*	G/G		A/G		A/A		G	A		
	108 (37.37%)	110.3	141 (48.79%)	136.5	40 (13.84%)	42.3	357 (61.76%)	221 (38.24%)	0.31	0.575
rs4680 *COMT*	A/A		A/G		G/G		A	G		
	75 (25.95%)	76.3	147 (50.86%)	144.4	67 (23.18%)	68.3	297 (51.38%)	281 (48.62%)	0.09	0.759
rs510769 *OPRM1*	C/C		C/T		T/T		C	T		
	147 (50.86%)	144.7	115 (39.79%)	119.6	27 (9.34%)	24.7	409 (70.76%)	169 (29.24%)	0.43	0.514

n—number of subjects.

**Table 5:** Cluster sizes and standardized distances between cluster centroids.

	Cluster 1 n = 95 (32.87%)	Cluster 2 n = 119 (41.18%)	Cluster 3 n = 75 (25.95%)
Cluster 1	0.000	2.013	2.011
Cluster 2	2.013	0.000	2.012
Cluster 3	2.011	2.012	0.000

n—number of subjects.

**Table 6:** Comparison of sex, smoking status, hypertension, and diabetes mellitus across the three identified clusters.

Variable	Cluster 1 (n = 95)		Cluster 2 (n = 119)		Cluster 3 (n = 75)		Χ^2^	*p*	*ϕ*	Power 1 − β
	yes	no	yes	no	yes	no				
Smoking status	17	78	4	115	10	65	12.37	0.002 *#	0.21	0.90
Hypertension	75	20	96	23	20	55	70.31	<0.001 *#	0.49	0.69
Diabetes mellitus	23	72	26	93	8	67	5.43	0.066	0.13	0.49
Sex	women	men	women	men	women	men				
	44	51	111	8	24	51	87.92	<0.001 *#	0.55	1.00

n—number of subjects; *p*—statistical significance; χ^2^—the chi-squared test; *—statistically significant difference, *p* < 0.05; #—Bonferroni correction: the *p*-value threshold was lowered to 0.0125 (0.05/4 = 0.0125).

**Table 7:** Comparison of anthropometric, hematological, biochemical, and quality-of-life parameters across the three identified clusters using one-way ANOVA.

Variable	Cluster 1 (n = 95) Mean ± SD	Cluster 2 (n = 119) Mean ± SD	Cluster 3 (n = 75) Mean ± SD	F_2,286_	*p*	η^2^	Power 1 − β
Age	67.72 ± 9.95	67.92 ± 9.60	63.69 ± 10.59	4.77	0.009 *	0.02	0.64
Body mass index (BMI)	30.14 ± 4.96	29.33 ± 5.70	27.93 ± 3.73	4.02	0.019 *	0.02	0.61
White blood cells (WBC, ×10^9^/L)	7.58 ± 2.11	6.54 ± 1.71	6.91 ± 2.02	7.82	<0.001	0.06	0.97
Lymphocytes (LYM, ×10^9^/L)	1.68 ± 0.49	1.64 ± 0.55	1.82 ± 0.68	2.27	0.104	0.01	0.41
Neutrophils (NEU, ×10^9^/L)	5.15 ± 1.92	4.23 ± 1.42	4.39 ± 1.64	8.92	<0.001 *#	0.07	0.99
Monocytes (MONO, ×10^9^/L)	0.56 ± 0.20	0.49 ± 0.16	0.53 ± 0.15	5.67	0.004 *	0.04	0.60
Eosinophils (EOS, ×10^9^/L) ǂ	0.13 ± 0.11	0.12 ± 0.10	0.12 ± 0.09	0.42	0.657	0.01	0.20
Basophils (BASO, ×10^9^/L) ǂ	0.04 ± 0.02	0.04 ± 0.02	0.03 ± 0.01	3.04	0.049 *	0.03	0.71
Hemoglobin (HGB, g/dL)	13.93 ± 1.59	13.28 ± 1.23	14.50 ± 1.22	19.10	<0.001 *#	0.10	1.00
Hematocrit (HCT, %)	41.70 ± 4.43	40.10 ± 3.39	42.41 ± 5.35	7.41	<0.001 *#	0.04	0.84
Mean corpuscular volume (MCV, fL)	91.47 ± 4.68	89.39 ± 4.51	91.63 ± 5.80	6.66	0.001 *#	0.06	0.96
Mean corpuscular hemoglobin (MCH, pg)	30.56 ± 1.95	29.52 ± 1.94	30.87 ± 2.22	12.42	<0.001 *#	0.08	0.99
Mean corpuscular hemoglobin concentration (MCHC, g/dL)	33.40 ± 1.02	33.11 ± 0.87	33.69 ± 0.93	8.96	<0.001 *#	0.04	0.88
Platelet count (PLT, ×10^9^/L)	257.36 ± 66.63	267.04 ± 75.49	237.47 ± 69.25	4.00	0.019 *	0.03	0.70
Platelet distribution width (PDW, %) ǂ	12.41 ± 1.74	12.29 ± 2.43	13.05± 3.23	2.26	0.104	0.02	0.50
Mean platelet volume (MPV, fL) ǂ	10.55 ± 0.80	10.46 ± 1.09	10.88 ± 2.22	2.14	0.118	0.02	0.50
Plateletcrit (PCT, %) ǂ	0.29 ± 0.19	0.27 ± 0.07	0.86 ± 3.19	3.50	0.032 *	0.03	0.69
C-reactive protein (CRP, mg/L) ǂ	4.02 ± 6.84	10.34 ± 32.33	8.43± 20.03	1.96	0.143	0.01	0.39
25-hydroxyvitamin D3 (ng/mL) ǂ	32.78 ± 15.87	33.41 ± 14.49	30.27 ± 18.83	0.91	0.404	0.01	0.27
Glycated hemoglobin (HbA1c, %) ǂ	5.83 ± 2.75	5.81 ± 0.93	5.56 ± 1.11	0.56	0.573	0.01	0.12
Creatinine (µmol/L) ǂ	84.61 ± 28.16	77.19 ± 27.51	78.72 ± 19.94	2.26	0.106	0.01	0.34
SF-36 PCS	43.27 ± 9.66	47.21 ± 9.96	45.83 ± 9.22	4.42	0.013 *	0.04	0.81
SF-36 MCS	53.16 ± 13.07	43.66 ± 13.46	47.69 ± 13.67	13.30	<0.001 *#	0.11	1.00

ǂ—variables do not follow a normal distribution according to the Kolmogorov–Smirnov test; n—number of subjects; SD—standard deviation; *—statistically significant difference, *p* < 0.05; #—Bonferroni correction: the *p*-value threshold was lowered to 0.002 (0.05/23 = 0.0022); F_2,286_—ANOVA F statistic.

**Table 8:** Sex-stratified comparison of anthropometric, hematological, biochemical, and quality-of-life parameters across the three identified clusters using one-way ANOVA.

	WOMEN					MEN				
Variable	Cluster 1 (n = 65) Mean ± SD	Cluster 2 (n = 53) Mean ± SD	Cluster 3 (n = 61) Mean ± SD	F_2,176_	*p*	Cluster 1 (n = 36) Mean ± SD	Cluster 2 (n = 39) Mean ± SD	Cluster 3 (n = 35) Mean ± SD	F_2,107_	*p*
Age	67.24 ± 10.78	63.86 ± 11.59	69.39 ± 8.27	4.00	0.020 *	68.57 ± 9.37	63.24 ± 9.03	68.33 ± 7.90	4.20	0.018 *
Body mass index (BMI)	29.46 ± 5.20	27.15 ± 4.50	29.61 ± 5.20	4.08	0.018 *	31.32 ± 5.41	28.33 ± 4.40	30.73 ± 4.84	3.75	0.027 *
White blood cells (WBC, ×10^9^/L)	7.07 ± 2.27	6.83 ± 1.75	6.62 ± 1.85	0.80	0.451	7.40 ± 1.95	6.68 ± 2.06	7.54 ± 1.47	2.20	0.116
Lymphocytes (LYM, ×10^9^/L)	1.58 ± 0.47	1.92 ± 0.61	1.56 ± 0.54	7.80	<0.001 *#	1.73± 0.43	1.75 ± 0.73	1.70 ± 0.48	0.07	0.937
Neutrophils (NEU, ×10^9^/L)	4.84 ± 2.12	4.24 ± 1.30	4.38 ± 1.58	1.92	0.150	4.90± 1.66	4.23 ± 1.71	4.96 ± 1.24	2.41	0.095
Monocytes (MONO, ×10^9^/L)	0.51 ± 0.17	0.48 ± 0.16	0.50 ± 0.17	0.54	0.585	0.57 ± 0.17	0.52 ± 0.12	0.66 ± 0.20	6.24	0.003 *
Eosinophils (EOS, ×10^9^/L) ǂ	0.10 ± 0.11	0.13 ± 0.10	0.12 ± 0.11	1.36	0.260	0.14 ± 0.09	0.10 ± 0.07	0.15 ± 0.12	2.60	0.079
Basophils (BASO, ×10^9^/L) ǂ	0.03 ± 0.02	0.04 ± 0.01	0.04 ± 0.02	1.47	0.233	0.04 ± 0.02	0.03 ± 0.02	0.04 ± 0.02	1.88	0.158
Hemoglobin (HGB, g/dL)	13.35 ± 1.15	13.58 ± 1.14	13.30 ± 1.15	0.89	0.413	14.28 ± 1.73	14.82 ± 1.07	14.44 ± 1.67	1.24	0.294
Hematocrit (HCT, %)	40.31 ± 3.25	39.92 ± 5.59	40.18 ± 3.35	0.13	0.876	42.57 ± 4.64	43.79 ± 3.23	42.84 ± 4.73	0.83	0.439
Mean corpuscular volume (MCV, fL)	90.10 ± 4.03	89.79± 5.79	90.36 ± 5.04	0.18	0.834	90.76 ± 4.75	91.45 ± 5.99	92.83 ± 4.08	1.48	0.233
Mean corpuscular hemoglobin (MCH, pg)	29.67 ± 2.12	29.86 ± 2.32	29.91 ± 1.94	0.21	0.812	30.41 ± 1.81	30.96 ± 2.12	31.31 ± 1.68	1.98	0.143
Mean corpuscular hemoglobin concentration (MCHC, g/dL)	33.11 ± 0.99	33.24 ± 0.98	33.10 ± 0.82	0.43	0.653	33.51 ± 1.03	33.86 ± 0.76	33.72 ± 0.87	1.40	0.250
Platelet count (PLT, ×10^9^/L)	266.97 ± 63.21	268.24 ± 67.27	269.05 ± 93.31	0.01	0.988	241.00 ± 48.94	233.73 ± 67.88	242.97 ± 65.33	0.22	0.799
Platelet distribution width (PDW, %) ǂ	12.30 ± 1.75	13.02 ± 2.65	12.07± 2.13	2.79	0.064	12.69 ± 2.24	11.49 ± 1.95	12.28± 1.48	3.61	0.030 *
Mean platelet volume (MPV, fL) ǂ	10.51 ± 0.83	10.70 ± 1.09	10.40 ± 1.08	1.23	0.294	10.61 ± 0.93	10.16 ± 0.95	10.48 ± 0.69	2.51	0.086
Plateletcrit (PCT, %) ǂ	0.30 ± 0.23	0.53 ± 1.75	0.27 ± 0.08	1.15	0.318	0.26 ± 0.05	1.12 ± 4.02	0.25± 0.06	1.56	0.215
C-reactive protein (CRP, mg/L) ǂ	6.84 ± 16.23	11.97 ± 35.54	5.91± 12.67	1.09	0.338	9.41 ± 35.64	8.04 ± 25.18	5.56± 9.89	0.19	0.827
25-hydroxyvitamin D3 (ng/mL) ǂ	36.20 ± 18.57	33.27 ± 16.97	33.16 ± 13.86	0.64	0.527	31.13 ± 11.40	27.85 ± 18.87	29.11 ± 15.04	0.41	0.665
Glycated hemoglobin (HbA1c, %) ǂ	5.87 ± 3.28	5.68 ± 0.44	5.81 ± 1.23	0.12	0.884	5.90 ± 0.83	5.73 ± 1.26	5.59 ± 1.08	0.72	0.491
Creatinine (µmol/L) ǂ	71.63 ± 15.03	69.24 ± 14.67	80.49 ± 33.74	3.73	0.026 *	98.40 ± 37.05	80.81 ± 14.69	90.51 ± 24.83	3.85	0.025 *
SF-36 PCS	42.82 ± 10.92	45.99 ± 8.74	48.58 ± 9.47	5.22	0.006 *	43.16 ± 9.13	45.29 ± 8.18	48.53 ± 11.00	2.77	0.067
SF-36 MCS	50.85 ± 14.94	46.30 ± 13.40	43.03 ± 11.78	5.15	0.007 *	54.10 ± 13.48	46.50 ± 13.50	47.35 ± 14.58	3.19	0.045

ǂ—variables do not follow a normal distribution according to the Kolmogorov–Smirnov test; n—number of subjects; SD—standard deviation; *—statistically significant difference, *p* < 0.05; #—Bonferroni correction: the *p*-value threshold was lowered to 0.002 (0.05/23 = 0.0022); F_2,286_—ANOVA F statistic.

**Table 9:** Comparison of genotype distributions for selected SNPs across the three identified clusters.

Variable	Cluster 1 (n = 95)			Cluster 2 (n = 119)			Cluster 3 (n = 75)			Χ^2^	*p*	ϕ	Power 1 − β
rs7975232 *ApaI*	C/C	C/A	A/A	C/C	C/A	A/A	C/C	C/A	A/A	124.65	<0.001 *#	0.66	1.00
	62	16	17	7	79	33	4	53	18				
rs2228570 *FokI*	C/C	C/T	T/T	C/C	C/T	T/T	C/C	C/T	T/T	35.25	<0.001 *#	0.35	0.99
	23	55	17	27	72	20	44	18	13				
rs1544410 *BsmI*	G/G	A/G	A/A	G/G	A/G	A/A	G/G	A/G	A/A	121.67	<0.001 *#	0.64	1.00
	76	6	13	22	81	16	10	54	11				
rs4680 *COMT*	A/A	A/G	G/G	A/A	A/G	G/G	A/A	A/G	G/G	4.15	0.386	0.12	0.33
	20	54	21	32	55	32	23	38	14				
rs510769 *OPRM1*	C/C	C/T	T/T	C/C	C/T	T/T	C/C	C/T	T/T	27.90	<0.001 *#	0.31	0.99
	53	31	11	44	68	7	50	16	9				

n—number of subjects; *p*–statistical significance; χ2–the chi-squared test; *—statistically significant difference, *p* < 0.05; #—Bonferroni correction: the *p*-value threshold was lowered to 0.01 (0.05/5 = 0.01).

## Data Availability

The data presented in this study are available on request from the corresponding author. The data are not publicly available due to privacy concerns.
